# Arsenious Acid in Apoplectic Congestions

**Published:** 1860-05

**Authors:** Lamarc Picquet

**Affiliations:** The Hospital of Honfleur


*Arsenious Acid in Apoplectic Congestions.* By Dr. Lamarc Picquet, of the Hospital of Honfleur.—The author gives as the result of his investigations on this subject the following conclusions: Apoplexy is essentially misunderstood, since the effusion of blood is only a secondary phenomenon. It is easy to master the preliminary symptoms of apoplexy, which is owing to an undue increase of blood globules. Arsenious acid is a valuable therapeutic resource in all congestions of the cerebral apoplectic form, since its first effect is to render the blood less rich in globules and less plastic. It is, however, indispensable, before the use of the agent, that the richness or condition of the blood should be determined, as, in case this fluid be poor in globules, the use of arsenious acid would increase such an abnormal condition.

The action of arsenious acid is connected so intimately with the digestive processs, that it should be employed while one is at the table, in order to facilitate its assimilation. The agent should also be used some time after the cure is effected, in order to increase the probability of the duration of the cure. The agent, however, cannot be considered absolutely antidotal: and hence the physician must always consider the mode of living, idiosyncrasies, and pathological condition of the patient. The dose is usually from one-sixteenth to one-sixth grain a day.

